# Musical development during adolescence: Perceptual skills, cognitive resources, and musical training

**DOI:** 10.1111/nyas.14911

**Published:** 2022-10-17

**Authors:** Daniel Müllensiefen, Paul Elvers, Klaus Frieler

**Affiliations:** ^1^ Department of Psychology Goldsmiths, University of London London UK; ^2^ Hanover Music Lab, University of Music, Drama, and Media Hannover Germany; ^3^ Datadrivers GmbH Hamburg Germany; ^4^ Max Planck Institute of Empirical Aesthetics Frankfurt a. M. Germany

**Keywords:** adolescence, intelligence, longitudinal study, music perception, musical development, working memory

## Abstract

Longitudinal studies on musical development can provide very valuable insights and potentially evidence for causal mechanisms driving the development of musical skills and cognitive resources, such as working memory and intelligence. Nonetheless, quantitative longitudinal studies on musical and cognitive development are very rare in the published literature. Hence, the aim of this paper is to document available longitudinal evidence on musical development from three different sources. In part I, data from a systematic literature review are presented in a graphical format, making developmental trends from five previous longitudinal studies comparable. Part II presents a model of musical development derived from music‐related variables that are part of the British Millennium Cohort Study. In part III, data from the ongoing LongGold project are analyzed answering five questions on the change of musical skills and cognitive resources across adolescence and on the role that musical training and activities might play in these developmental processes. Results provide evidence for substantial near transfer effects (from musical training to musical skills) and weaker evidence for far‐transfer to cognitive variables. But results also show evidence of cognitive profiles of high intelligence and working memory capacity that are conducive to strong subsequent growth rates of musical development.

## Graphical abstract

Quantitative longitudinal studies on musical and cognitive development are very rare but can offer insight into causal mechanisms driving the development of musical skills and cognition. This paper documents longitudinal evidence from three different sources: a systematic literature review, a model of musical development from a large birth cohort study, and data from the LongGold project. Results suggest substantial near transfer effects and weaker far‐transfer effects on cognitive variables.

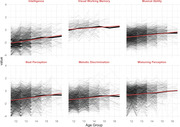

## INTRODUCTION

Adolescence^a^ is a decisive period in human development where neuro‐plasticity is high, many cognitive skills are acquired,^1^ important socioemotional changes take place, and self‐identities^2^ are formed. For many individuals, adolescence is the period that includes a conscious and self‐directed choice to engage with music intensively and devote personal resources to instrumental practice and music playing (or not).^3^ The musical choices individuals make during adolescence often set the path for the type and intensity of engagement with music across a lifetime.^4^ At the same time, adolescence can be an important period of development for cognitive resources, such as working memory or general intelligence, and opportunities for cognitive growth through external stimulation (e.g., musical or other forms of specialized training), which are considered highly important.^5^

Despite its crucial role, the empirical evidence documenting musical development during adolescence is very scarce. This is one severe disadvantage for studies trying to understand the relationships between musical training and the development of important cognitive resources, such as working memory and intelligence. In the past, many studies investigating the so‐called transfer effects of musical training on the development of cognitive skills and resources have almost exclusively considered far‐transfer effects from musical training to a nonmusical domain.^6^, ^7^, ^8^, ^9^ But there is mounting evidence that near‐transfer effects (i.e., the effect of musical training on perceptual musical skills that are not the primary target of the training intervention) are crucial for understanding the mechanisms by which musical training^10^ can have an impact on skills and resources outside the musical domain. Hence, even if the primary interest of a study is on far‐transfer effects, ignoring the development of musical abilities and skills might lead to an incomplete picture of the mechanisms and processes that relate to musical training and cognitive development. Phrased differently, including the development of musical skills and abilities into the modeling of this relationship could potentially help to resolve the conflicting findings that are frequently reported in this research area.

Hence, the present paper has two main goals. The first goal is the documentation of available empirical evidence for musical development across childhood and adolescence from longitudinal studies. This aims to provide the empirical background for further studies on musical development. We consider individual research studies with and without music interventions in a systematic review. In addition, we also model data from a general longitudinal study, the British Millennium Cohort Study (MCS). The MCS does not have a specific music focus but does include several items that are related to musical behavior, engagement, and abilities, and together with its large sample size and its representativeness (for the British population) thus allows valuable insights into musical development in the general population.

The second goal of this paper is the modeling of musical development together with the development of fundamental cognitive resources (i.e., general intelligence and working memory) using data from an international longitudinal study, the LongGold project. Modeling these longitudinal data can provide novel insights into the relationship between musical and cognitive development while also considering the amount of musical training that an individual receives during adolescence.

### Investigating the relationship between cognitive resources, musical training, and musical skills

Working memory and general intelligence are domain‐general resources that are underlying many cognitive processes and are closely connected to the development and the use of complex intellectual abilities,^11^, ^12^ including musical listening skills and instrumental learning.^13^, ^14^ Indisputably, musical training is a necessary requirement for the development of musical motor skills and instrumental learning. However, the traditional debate on whether the development of fundamental cognitive resources, such as working memory or general intelligence, is a prerequisite or a consequence of musical training has intensified recently.^15^, ^16^ On one hand, there is evidence suggesting that working memory capacity and executive functions increase in response to musical training^17^ through music‐induced brain plasticity.^18^ On the other hand, working memory and general intelligence are described as necessary components of the cognitive profile of successful music learners that may be largely determined by genetics.^19^, ^20^ As Silas et al.^16^ have shown recently, cross‐sectional data can be helpful to narrow down the set of possible causal hypotheses but only under certain conditions can cross‐sectional data actually provide evidence for just a single causal model consistent with the data.

In contrast, definite answers to causal questions are usually expected from experimental studies with random assignment of participants to a music training versus nonmusic training group.^13^, ^17^, ^21^ Here, random assignment helps to match both experimental groups in terms of any variables that might otherwise confound the effect of musical training. This is because, given a sufficiently large sample, any association between confounding variables and musical training will be removed if participants are assigned randomly to the experimental conditions differing by the degree of musical training. After random assignment to different intervention groups, cognitive or musical skills need to be assessed and compared at later time points after musical training could have potentially affected the development of musical and cognitive skills.

While the randomized control trial (RCT) methodology is appealing due to its conceptual simplicity, it also has a number of practical and limiting drawbacks in our case. The RCT approach requires a clear distinction between the experimental groups in terms of the musical training received. However, it is difficult to ban participants in the control group for extended time periods from receiving any musical training. Hence, many music training intervention studies are limited to relatively short time periods (e.g., from 6 months to 2 years). Another drawback of the RCT approach is that results are often difficult to generalize because musical training interventions often rely on specific music training programs provided by collaborating institutions, which makes the effects of interventions difficult to compare across studies.

### The LongGold project

The LongGold longitudinal study has chosen an approach that is deliberately different from and complementary to RCT studies investigating the effects of musical training. Instead of assigning participants to different groups and administering a musical intervention, the “naturally occurring” musical activity and training of participants is observed and recorded at regular intervals across the duration of the study. Additionally, musical skills and general cognitive abilities are recorded at the same time intervals. The resulting longitudinal data can be used to model developmental trajectories for all three constructs (cognitive resources, musical skills, and musical training) and causal relationships can potentially be revealed through the difference in developmental changes over time on these variables. For example, one participant might start intensive musical lessons at some point during adolescence, but their “statistical twin” (i.e., a participant with a very similar psychological and skills profile except for differences in music training) would not increase their levels of musical activity and training. The comparison of the developmental trajectories for music perception and cognitive skills of these statistical twins can enable causal inference on the effect of musical training. Thus, in general, the design of the LongGold study aligns closely with study designs in educational or economic research where researchers are not able to manipulate independent variables but have to infer causal effects from the change in economic or educational policies that are beyond their control (see good introductions to causal inference for typical scenarios in these domains^22^, ^23^, ^24^).

The LongGold study uses a longitudinal design where the same secondary school students are assessed on a battery of performance tests and self‐report questionnaires every year. The battery comprises performance on different music perception skills, including melodic discrimination, beat perception, intonation perception, rhythm processing, musical emotion discrimination, melodic imagery ability, and harmony perception. In addition, cognitive performance capacities (general IQ and working memory), personality, and psycho‐social skills are assessed as well. Finally, school grades are collected for all participating students every year. The study started in 2015 with a single school from the UK, but in subsequent years, 10 schools from different regions in Germany and the UK have been participating. The overall goal of the study is the documentation of the development of important musical as well as cognitive and psycho‐social variables from the beginning to the end of secondary school, broadly covering the 10–18 years range. The study is still ongoing and the current paper, therefore, presents only preliminary results. Study goals and initial cross‐sectional results are described in Müllensiefen et al.^25^

### The present study

The present study consists of three parts that provide independent evidence for the development of musical skills across childhood and adolescence and their relationship with indicators of musical training and engagement as well as measures of general cognitive resources. The three parts make use of very different datasets: (1) a systematic review of published papers, (2) an omnibus study on human development, and (3) a specialized study on musical development. The three parts report different constructs and cover varying age ranges. However, the common feature of all three parts is the focus on the development of musical skills during adolescence and the use of longitudinal data.

Our emphasis is on (1) quantitative data from objective performance tests of musical ability, (2) longitudinal data from the same individuals, and (3) data from children and adolescents from the general population. The emphasis on these three aspects makes the present study comparable to studies documenting the development of general cognitive abilities, such as fluid and crystalized intelligence^26^ or working memory capacity,^27^ where typical growth curves and norm data can help to inform educational training, clinical interventions, or cognitive research. To our knowledge, no comparable longitudinal datasets on musical skills in adolescence exist in the published literature. The emphasis on these three aspects also distinguishes the present study from related research on musical development in other studies that is primarily based on qualitative data, such as interviews,^28^, ^29^, ^30^, ^31^ biographical information of musicians,^32^ or individuals receiving specialized music education.^33^ Finally, obtaining true longitudinal data from the same individuals through repeated testing is different from cross‐sectional data that is stratified by age.^34^ Longitudinal data allow for different types of inference and give rise to different developmental curves compared to cross‐sectional data, as has been shown in studies comparing longitudinal and age‐stratified cross‐sectional data.^35^, ^36^, ^37^

The systematic review in part I summarizes empirical results from longitudinal studies on musical skills and abilities in the published literature. In part II, data on musical constructs made available through the MCS are modeled with a focus on the interplay of musical abilities and musical engagement. Finally, part III presents the first preliminary analysis of longitudinal data from the LongGold project and specifically addresses how musical development relates to musical training and the development of general intelligence and working memory.

### Part I: Systematic review of longitudinal studies on musical development

A systematic literature review was conducted in August 2017 to gather all published studies that assessed musical abilities in a longitudinal study design. Hence, this review is different from the review by Ilari^38^ that targeted longitudinal studies on music education and child development, mainly reporting development on cognitive, psycho‐social, or educational measures. Our procedure followed the guidelines of the PRISMA Statement.^39^ Our aim was to identify studies that: (1) assessed musical abilities behaviorally, either as a music perception or production task using a quantitative measure; (2) provided at least two measurements of musical ability from the same individuals; (3) used time intervals between measurements that were sufficiently large in order to track developmental changes (minimum duration of 4 weeks); (4) covered developmental changes during childhood and adolescence (i.e., participants between 3 and 20 years of age); and (5) provided descriptive statistics (i.e., means and standard deviations) on measures of musical ability at each time of measurement. Studies that only investigated neural processes or studies that provided cross‐sectional evidence were excluded. Due to the general aim of identifying the development of musical abilities in the general adolescent population, special populations, such as those with learning disabilities (e.g., dyslexia) or developmental or clinical disorders (e.g., autism), were excluded.

In order to identify candidate publications, the following four scientific search engines and indexing services were employed: PubMed, Scopus, PsycINFO, and Web of Science. We ran keyword searches for “music” and “abilit*” or “skill*” or “expertise” and “development*” or “longitudinal.” Search results matching these criteria comprised 4997 entries which were reduced to 3236 studies after duplicates were removed. Each of the 3236 publications was assessed for eligibility based on title and abstract. The full text of 38 publications was then assessed in a subsequent step, excluding 33 studies that did not match the inclusion criteria. The remaining five studies were examined for quantitative synthesis. One study^40^ that met the inclusion criteria was published just after the systematic literature search was conducted and was, therefore, added to the set of reviewed studies later. See Table S1 in the supporting material for detailed descriptive information on the selected studies.^b^

## RESULTS

### Description of studies included for quantitative synthesis

The publications by Hassler^43^ and Hassler and Birbaumer^44^ are based on the same longitudinal study but focus on different grouping factors (musicality vs. gender) and provide different longitudinal information. Hence, they are processed separately and displayed as two different studies in the following results.^c^ The main aim of their longitudinal studies was to examine the development of musical abilities along with other cognitive variables, such as visual and spatial abilities and verbal fluency. The study duration was 8 years with yearly measurement intervals and involved 120 participants. For the assessment of musical abilities, the Wing's battery (tests 1–3) of musical abilities was employed.^47^ As one of her main findings, Hassler^43^ observed that while visual, spatial, and verbal abilities increased roughly linearly during the study (covering the age span from around 11 to 18 years of age), the developmental trajectory of musical abilities showed no general positive trends over the course of the longitudinal study. Additional findings indicated that participants who regularly engaged with music (either by composing or improvising) performed overall better on tests of musical abilities but exhibited a similar horizontal development as participants without regular active music engagement. Hassler^43^ identified gender differences in the development of musical abilities, revealing a tendency for higher performance among male participants.

Ilari and colleagues^48^ published results from a longitudinal study spanning 1 year with one measurement point at the start of the year and another at the end of the year. The primary goal of their study was the assessment of the effectiveness of an El Sistema‐Inspired music education program. The data from participants receiving this treatment program were compared to participants in a control condition. For the assessment of musical abilities, Gordon's Primary Measures of Music Audiation were employed (PMMA).^49^ Fifty participants from 6 to 7 years of age were observed. The study revealed a positive general trend in musical abilities across conditions and a stronger improvement on the tonal discrimination test in the treatment condition as compared to the control. This finding seems to contradict Gordon's assumption that individuals would generally differ in their absolute levels of musical aptitude but show very comparable increases in test scores over time. However, no significant differences between conditions were reported for the PMMA rhythm test.

Yang and colleagues^50^ investigated the relationship between musical abilities and cognitive development in nonmusical domains. More specifically, the study inquired whether musical skills predict the development of first and second language learning as well as in mathematics. Musical abilities were assessed with a self‐designed test that assessed “music pitch identification, melody representation, and singing from semester 2 to 5.”^50^ Concerning the development of musical abilities, Yang and colleagues describe a general developmental trajectory that begins with a decrease in musical abilities from age 7 to 8. After this initial decline, musical abilities plateau in the group that does not receive additional musical lessons until the age of 11.5. In the group that received additional training, musical abilities steadily increased over the course of time with an increased growth at age 10.5. While both groups showed very similar musical ability levels at the beginning of the study, after 4.5 years, there was a considerable difference between the groups.

Cohrdes and collaborators^40^ investigated the development of musical abilities in children during the last year in kindergarten. Two hundred and two participants were around 5.5 years of age at the start of this 1‐year‐longitudinal study with two measurement points. The primary aim of this study was to investigate the development of musical abilities and to identify external contributing factors. The study employed a formal music training intervention as well as two control conditions. Along with the PMMA, several other measures of musicality assessed skills in rhythm, synchronization, and emotion recognition. Cohrdes et al. found a general positive developmental trend across experimental conditions, with a significantly steeper increase in tonal discrimination, rhythm repetition, and synchronization skills for the musical training intervention group compared to the passive control group. However, the only significant difference in performance growth between the music intervention and the active control group was for the rhythm repetition task.

### Quantitative synthesis of developmental trends

**FIGURE 1:**
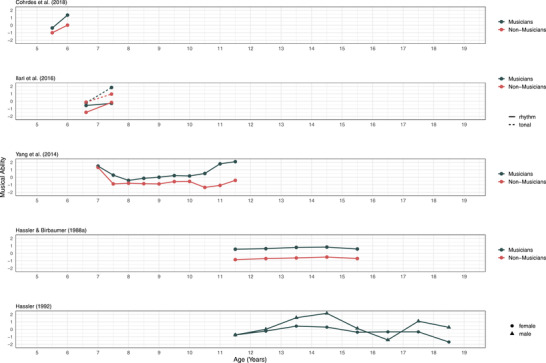
Longitudinal assessment of musical abilities. Comparison of findings from five different longitudinal studies. Note that all data from dependent variables (y‐axes) have been z‐transformed to enable easier comparisons across the different studies. Higher values indicate better performance on the musical ability tests used. Also, note that “nonmusicians” refer to participants without formal music instruction or specific musical intervention.

Because the five studies reviewed above employed different measures of musical abilities, scores could not be compared directly. The analytic strategy, therefore, was to visually interpret the developmental curves provided by each study by arranging them on a developmental grid. Since all studies provided information on the age of participants, it was possible to align the curves on the x‐axis by the age and standardize (i.e., z‐transform) the ability or achievement scores of each study such that all studies could be displayed using the same scale (ranging from −2 to 2). Figure 1 displays developmental trends of musical abilities as assessed in the five selected longitudinal studies. Together, the studies cover a developmental range from 5.5 to 18.5 years of age. Each individual line represents the average scores for one group of participants according to a grouping factor within each study. The grouping is based on the level of music instructions that participants receive before or during the study, on gender, or the type of measure used. In summary, the developmental data gathered here suggest that formal musical instruction improves musical abilities, especially in childhood and that the increases in musical abilities are larger in earlier childhood compared to later adolescence. Differences between musically active and musically passive individuals appear then to remain stable throughout adolescence.

Despite these interesting trends that the quantitative synthesis reveals, the generalizations drawn from the systematic review are limited in several ways. First, three studies^43^, ^44^, ^50^ provided descriptive statistics of musical abilities only visually (i.e., line plots indicating longitudinal development). In order to still make use of the data, the values were estimated by graphically measuring the distances on the graphs in the publications. Second, studies differed with regard to the type of music intervention as well as how the distinction between musically active and nonactive individuals was drawn. These methodological differences may be partly responsible for the difference in developmental trajectories visible in Figure 1. Third, and most importantly, the assessments of musical abilities in these five studies employed different tests and assessment procedures, which makes it difficult to know to what degree changes in ability are due to the measurement instruments and to what degree results can be generalized. Fourth, the tests employed may not have been suitable or specifically designed to observe developmental trends. For example, the music achievement test used by Yang and colleagues^50^ changed across the longitudinal study and incorporated additional music theory components that were not part of the earlier measurements. This makes it difficult to determine whether participants did not improve their skills or whether the test had become more difficult.

In sum, the systematic review of longitudinal studies on music development revealed some empirical trends on the effect of age and musical training on musical abilities. However, the discussed limitations and the small number of studies that met the inclusion criteria of the review make it difficult to draw valid and reliable generalizations on the development of musical skills and abilities. This clearly demonstrates how little quantitative data on musical development are available in the published literature and highlights the need for further longitudinal studies. Though, it is worth noting that several longitudinal studies^18^, ^42^, ^51^ on musical development are currently ongoing with new results likely to be available in the near future.

### Part II: Modeling music‐related data from the MCS

Two of the main limitations of the studies reviewed in part I are their small sample sizes and the difficulty to generalize results to a larger population. These difficulties are addressed in part II where we analyze data on musical engagement and abilities from a very large longitudinal study that can be considered representative due to its efforts to include all babies born in the UK during a specific time period.

## METHODS

The MCS^52^ is a longitudinal cohort study of children (“Millenials”) in the UK born at the beginning of the 21st century (between September 2000 and January 2002). The infant sample was drawn from child benefit registers, a welfare payment that was available for nearly all families in the UK and was, therefore, considered an approximately exhaustive register of all newborns in the UK. The initial sample at 9 months of age comprised 18,818 children and their respective parents. They were followed by further surveys at ages 3, 5, 7, 11, and 14 years, totaling six survey waves. Missing values were assumed to be missing at random.^d^

Because the MCS does not assess musical abilities with a standardized measure, proxy measures available in the data were selected. All waves were screened for potential music‐related variables. Ten music‐related variables were identified in waves 2–6. The data from the five surveys containing musical variables were merged based on the unique household identifier. Families with twins (*n*  =  246) or triplets (*n*  =  10) were removed to facilitate data merging. Four music‐related variables (EPMUSL00, EPMUSC00, cpplmub0, and cmplmub0) were not considered due to the high number of missing values (> 9000).

The following six variables were used in the final analysis: (1) at 9 months of age, parents were asked “How often do you teach your child songs/poems/rhymes?,” ranging from “Occasionally or less than once a week” to “7 times a week constantly” on a 7‐point scale (variable ID: bmofsoa0); (2) at 7 years of age, cohort members were asked “How much do you like listening or playing music?” ranging from “I like it a lot” to “I don't like it” on a 3‐point scale (variable ID: dcsc0001); (3) at 7 years of age, teachers were asked to evaluate cohort members’ abilities in “Expressive and Creative Arts (e.g. art & design, music),” ranging from “Well above average” to “Well below average” on a 5‐point scale (variable ID: DQ2170); (4) at 11 years of age, cohort members were asked “How often do you listen to or play music, not at school?,” ranging from “Most days” to “Never” on a 5‐point scale (variable ID: ECQ01×00); (5) at 11 years of age, teachers were asked to evaluate cohort members’ musical abilities, ranging from “Well above average” to “Well below average” on a 5‐point scale (variable ID: EQ2F; and (6) at 14 years of age, cohort members were asked “How often do you listen to or play music, not at school?,” ranging from “Most days” to “never or almost never” on a 6‐point scale (variable ID: ECQ01×00). These variables represent proxy assessments of what is best characterized as musical engagement, except for the teacher evaluations at 7 and 11 years of age, which will be considered as proxy measures of musical ability.

## RESULTS

We employed structural equation modeling using the software package “lavaan”^53^ in R^54^ to identify developmental trends in musical abilities. The aim was to model the influence of earlier assessments of musical engagement and abilities on subsequent assessments and to investigate which variables at which time periods would best predict musical engagement and abilities during later stages. Following suggestions by Hayduk and Littvay,^55^ we used single indicator variables to separate the measurement from the structural model of regression and covariances. Different models were fit using robust maximum likelihood estimation and the full information maximum likelihood method for handling missing values. Model fits were compared on the Bayesian information criterion (BIC) and due to the large sample size (*n* =  18,980) and the corresponding high statistical power to detect significant, but very small and practically irrelevant relationships, a significance threshold of *p* < 0.001 was set for evaluating the significance of individual path coefficients.

**FIGURE 2:**
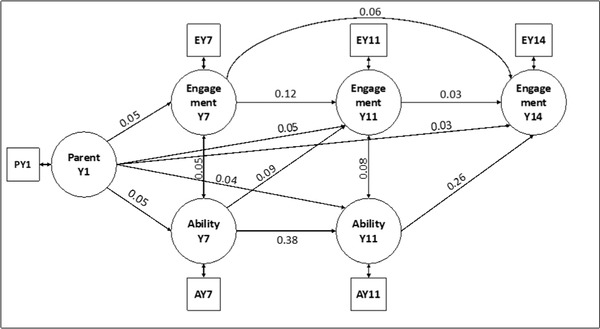
Structural equation model of Millennium Cohort Study data displaying only significant (*p* < 0.001) regression paths with standardized coefficients (*n* = 18,980).

In the first step, a fully saturated model was fitted (BIC  =  196,945), which had two regression paths with *p*‐values above the specified threshold. Both paths were removed, and the model was refit. The revised model showed an improved fit (BIC  =  196,939) and only contained regression and covariance coefficients with associated *p*‐values below the specified threshold. The model's comparative fit index was at 0.992 and the Tucker–Lewis Index at 0.942, both indicating very good model fits. Therefore, the model was accepted as the final model and is displayed graphically in Figure 2. Standardized beta coefficients are displayed next to the directed arrow representing the corresponding effect. Effects above the specified significance threshold are not displayed in the figure.

The analysis of longitudinal data from the MCS identified significant positive relationships between musical abilities at all measurement stages during childhood. The relative strength of the individual effects can be understood by comparing their standardized regression coefficients (*β*). The strongest predictor for a musical engagement at age 14 was the teacher's music skill assessment at age 11 (*β* = 0.24). Additionally, the teacher's assessment of artistic skills (including music) at age 7 (*Ability Y7*) was the strongest predictor of musical ability at age 11 (*β* = 0.37). In turn, musical engagement at year 7 and year 11 had weaker effects on musical engagement assessed at year 14. Musical parent–child interactions at 9 months (Parent Y1) only had a small influence on children's engagement and ability at later time points. Taken together, the results show that the level of artistic and musical skills (*Ability Y11*) had a stronger effect on musical engagement during adolescence (*Engagement Y14*) than musical engagement at earlier stages (*Engagement Y7* and *Engagement Y11*). Early musical parenting activities only had a small but significant effect on musical skills assessed at later stages, similar to the positive effects of an enriched musical home environment of 2‐ to 3‐year‐old children that have been reported in the literature.^56^ However, a limitation of these results generated from MCS is that all measurements of musical constructs are exclusively derived from self‐reports or are coming from reports provided by teachers and parents. Hence, while omnibus studies like the MCS can provide data from large and representative samples, the quality and comprehensiveness of the musical measurements is typically low compared to studies with a dedicated music focus.

### Part III: The development of musical skills, working memory, and general intelligence during adolescence

Part III presents an analysis of the data collected on the LongGold study which relates musical development to the development of working memory and general intelligence as general cognitive resources.^e^

## METHODS

### Participants

The LongGold study is still ongoing and data from 4,333 participants are used for the current analysis. In this preliminary dataset, the mean number of times that a participant has taken part in the study participation is 2.6 (SD  =  2.6). The mean age at study entry is 11.8 (SD  =  1.7; range = 9–17) years of age, and the mean age at the latest study participation is 12.7 (SD = 1.6; range = 9–17) years of age. 57.2% self‐reported as females and 35.8% as males (7% of responses were “other” or declined to provide gender information). Data were collected between 2015 and 2020 at five schools from the southeast of England and eight schools from different regions in Germany.

### Design

Different schools joined and left the study at different points in time. Most schools joined the study following a cohort‐sequential design where children enter the study in their first year of secondary school and participate in annual testing sessions until their planned exit at the end of their secondary school time. However, recently, schools that joined the study follow a more efficient accelerated design where several year groups at the same school enter the study simultaneously and each year group only participates for only 4 years. The dataset used for this analysis also contains data from schools that participated for less than the planned number of years due to administrative or logistical difficulties, and it comprises data from students who only participated once and dropped out subsequently (e.g., due to changing the school, temporary illness, revoked study consent, etc.). Nonetheless, data from participants for whom only single measurements are available are still useful for estimating the variance at individual time points.

### Stimulus and materials

More than 25 different tests and questionnaires have been used as part of the LongGold project over the years. See a more comprehensive description of the measures employed in the project^25^ and the project's open and free web resources.^f^ However, in line with the focus on musical and cognitive development of this paper, this analysis only makes use of data from seven measures that represent either general cognitive resources, music performance skills, or aspects of musical training and engagement.

#### Jack & Jill working memory test (JAJ)

The Jack & Jill working memory test (JAJ)^60^ measures visuospatial working memory capacity by employing a dual‐task paradigm. The task is adaptive and based on an explanatory item response theory (IRT) model. The final IRT scores of the test show a bell‐shaped distribution that is centered around 0. Negative scores indicate a performance worse than the average of the calibration sample and positive scores indicate a better than average performance. The test is implemented in the open‐source R package JAJ.^g^ The test length on the LongGold project has been set to eight trials. The JAJ has shown good reliability (marginal reliability = 0.8; standard error of measurement [SEM] = 0.27 SDs) and correlational validity with related cognitive constructs, such as general intelligence, mechanical reasoning, or shape rotation (Pearson *r*‐values between 0.46 and 0.57).^60^

#### General intelligence (MIQ)

General intelligence was assessed using the MIQ test,^61^ a matrix reasoning test modeled on the Raven's progressive matrices. Similar to the JAJ working memory test, the MIQ is adaptive and the difficulty of the visual matrices increases when participants give correct answers. The test was developed by the Cambridge Psychometrics Centre, as part of the International Cognitive Ability Resource project, and items have been validated against the copyrighted version of Raven's matrices. Each item is timed to a maximum of 2 min, after which the target matrix disappears and participants have to make a choice to progress to the next item. The length of the test is set to eight items. The reported internal consistency of the MIQ progressive matrices can be considered good (Cronbach's α = 0.89, MacDonald's ω = 0.84) and its correlational validity has been established determined by comparison to general school achievement tests (*r* = 0.372; *p* < 0.001).^62^

#### Beat perception ability (BAT)

Identifying the musical beat is a fundamental ability that is part of many processes in music perception and production,^63^ and the individual ability of beat identification and processing can be measured using an adaptive version of the beat alignment test (BAT).^64^ On each trial of the BAT, participants are presented with two versions of a naturalistic musical track (drawn from popular music genres), both overlaid with a metronomic click track. The ON version of the track has the click in time with the musical beat locations, while the OFF version has the probe track displaced away from the musical beat locations. The participant's task is to identify the ON track. Across the different waves of data collection on the LongGold project, the BAT was administered with 18, 20, or 22 trials, and participant scores were computed using an underlying IRT model.^64^ Reliability (SEM  =  0.67; test‐retest correlation  =  0.67) and correlational validity with self‐report measures of musical training (*r*  =  0.41) of the BAT are in the acceptable to good range.^64^

#### Melodic discrimination ability (MDT)

Melodic discrimination ability is another very fundamental musical skill that enables the recognition and structuring of musical material in melodic music. Melodic discrimination ability was measured using the adaptive test MDT.^65^ This test uses a 3‐AFC response task with each item consisting of three versions of a melody at different transpositions in pitch. Two of these versions are identical and one is different. The participant's task is to identify the nonidentical melody while ignoring transpositions between versions. Across different test waves, the length of the adaptive MDT differed slightly and ranged from 18 to 20 trials. Reliability (SEM  =  0.62) and correlational validity with the Musical Ear Test and a test of musical imagery ability (*r*  =  0.52 and *r*  =  0.57) of the MDT are in the acceptable to good range.^16^, ^65^

#### Mistuning perception ability (MPT)

The Mistuning Perception Test (MPT)^66^ measures the ability to perceive differences in tuning or intonation between a lead voice and the instrumental background track in popular music. Being able to perceive the “in‐tuneness” of a singing voice is of great importance for the development of singing skills and for judging the quality of musical performances. On each trial of the MPT, two variants of a short excerpt from a pop music song are presented where one variant has the entire voice track pitch‐shifted in relation to the background track. Participants have to identify the pitch‐shifted version which is described as the one where the singer sings out of tune. The explanatory IRT model that is underlying the MPT relates perceptual difficulty to the amount and direction (i.e., up vs. down) of pitch‐shifting. The MPT has been used with 18, 20, or 22 trials in different LongGold test waves. Reliability (SEM  =  0.52; test‐retest correlation  =  0.7) and correlational validity with a test of pitch discrimination ability (*r* > 0.5) of the MPT are in the acceptable to good range.^66^

#### Self‐reported musical sophistication (Gold‐MSI)

The Goldsmiths Musical Sophistication Index (Gold‐MSI)^67^ is a self‐report inventory measuring musical skills, expertise, and sophistication in music‐related behaviors. It comprises five subscales focusing on active musical engagement, self‐reported music perception abilities, musical training, singing abilities, and sophisticated emotional use of music. For the current paper, only the data of the musical training subscale are presented. Scale scores range between 1 and 7. The musical training subscale assesses the amount of formal musical training and practice as well as achievement across a participant's lifetime. Hence, the way that items are phrased in the subscale is designed to measure a stable construct rather than a momentary state that could see frequent changes. Reliability of the musical training subscales is high (Cronbach's alpha  =  0.9; MacDonald's omega  =  0.9; test‐retest correlation  =  0.97) and correlational validity with the Advanced Measures of Musical Audiation is good (*r*  =  0.43).^67^

#### Concurrent musical activity (CCM)

Contrasting with the Gold‐MSI musical training subscale, the Concurrent Musical Activity questionnaire (CCM)^25^ is a short self‐report instrument that measures momentary levels of musical activities, practice, and music‐making during the past 3 months. Especially during school years, musical activities and practice levels can change suddenly and frequently, for example, when certain musical activities are offered or not offered anymore as part of the school curriculum or when life circumstances change or adolescents set new priorities regarding their leisure activities.^3^ Scores for the CCM are computed using IRT scoring and principal component analysis and range between −3 and 9.

### Demographics

A brief demographic questionnaire asked participants about age, gender, nationality, languages spoken, handedness, and any hearing impairments.

### Procedure

Data reported in this publication were collected between 2015 and 2020. Participants were tested annually around the same time of the year. Testing was carried out in groups of 10–25 participants. Participants were tested using an online test battery that was implemented with the psychTestR package^68^ within the Shiny framework for R. Each participant accessed the online test battery via their own desktop or tablet computer and using a provided pair of headphones (Sennheiser HPM 1000, Sennheiser electronic GmbH & Co. KG, Wedemark, Germany). Participants worked through the online test battery in silence and at their own pace. Testing took place during school hours and the completion of the test battery took generally between 50 and 90 min, depending on the age and the reading speed of the participant, as well as the number and type of tests and questionnaires selected in a particular testing wave. Testing sessions were normally supervised by one or two research assistants and the class teacher. Exceptions were made during 2020 when, due to the COVID‐19 pandemic, most test sessions had to be carried out without the presence of research assistants or were joined from home.

The study obtained ethical approval from the ethics committee at Goldsmiths, University of London and from the research ethics committee at the Leibniz University at Hannover, Germany. In addition, the study was approved by the ministries of culture and education of the German federal states of Hesse, Baden‐Württemberg, and Bavaria. Study participation for students was completely voluntary. In addition, study consent was sought from parents in advance of the first wave of data collection prior to each new wave of data collection, depending on the requirements of the school and the stipulations of the governmental approval obtained.

### Data analysis strategy

The analysis of the LongGold project data follows the approach described by McArdle and Nesselroade.^69^ They suggest a five‐step approach for analyzing the change in longitudinal data.

- Step 1 considers developmental trajectories or *intraindividual change*. From the LongGold data, we can answer the question of how musical abilities, general intelligence, and working memory develop across the teenage years, that is, determine the growth in performance scores per year and whether growth rates appear to change or stay approximately linear across the age range considered.
- Step 2 focuses on differences in developmental trajectories or *individual differences in intraindividual change*. This step can demonstrate how much adolescents differ in their development.
- Step 3 investigates the codevelopment of trajectories for different skills or cognitive resources and targets *interrelations in behavioral change*. Without assuming a causal direction, this step asks how development in different areas coevolves.
- Step 4 aims to identify variables that can potentially explain developmental trajectories, that is, *causes of intraindividual change*. Here, the central research question asks for external factors that drive development.
- Step 5 investigates variables that explain differences in developmental trajectories or *causes of interindividual differences in intraindividual change*. Thus, this step examines why some individuals develop differently from others.

While steps 4 and 5 might provide answers to the seemingly most interesting questions, addressing all five steps is nonetheless necessary to provide the background and empirical overview of adolescent development of musical and nonmusical skills and resources. Hence, the data can be used for comparisons with other longitudinal datasets, such as the ones presented in parts I and II. In addition, the developmental models across the five steps provide a useful background for music educators and developmental researchers in that they make general trends visible against which individual developmental trajectories can be compared.

The analytical approach suggested by McArdle and Nesselroade^69^ can be implemented with different statistical frameworks. In particular, structural equation and multilevel modeling are well‐suited for this task and can produce near‐identical results for many analysis scenarios.^70^ For the current paper, we will primarily make use of the multilevel approach via mixed effects models. Mixed effects models have the advantage that they are widely used and understood within psychology and are often easier to specify. This comes at the cost of reduced flexibility, for example, in terms of incorporating known measurement error in independent as well as dependent variables or complex covariance structures. Hence, future analyses of the LongGold data might take advantage of the greater flexibility of the structural equation modeling approach.

## RESULTS

### Data processing

For the following analyses, we chose *age group* (i.e., the school year group) as a time variable rather than chronological age because this yields approximately equal distances between measurements and exactly one set of observations per time unit for each participant. Values on the time variable are relabeled to reflect the average age (rounded to half‐year steps) of the age group.

In addition to considering the three tests of musical abilities (MDT, BAT, and MPT) individually in the following analyses, we also compute scores of the latent variable *general musical ability* using the three tests as indicator variables in a factor analytic model. The factor model showed metrical (i.e., weak) and scalar (i.e., strong) invariance across the different testing waves (all *p* > 0.3) when tested through confirmatory factor analysis. Hence, the variable *general musical ability* appears to be suitable for comparisons across time. However, during the first three waves of data collection, only the MDT and the BAT were employed as tests of musical listening ability, and scores of these two tests were averaged. To ensure the comparability of these simple averages and factor scores, we compared averaged MDT and BAT scores with the scores extracted from the factor model using all three tests as indicator variables for data collection waves 2018–2020. This indicated very high correlations (all Pearson correlation coefficients > 0.97) for all 3 years. Thus, we decided to use averages of all three measures for all waves as a proxy for the factor scores of the latent variable *general musical ability*.

**Step 1. Intraindividual change**

**FIGURE 3:**
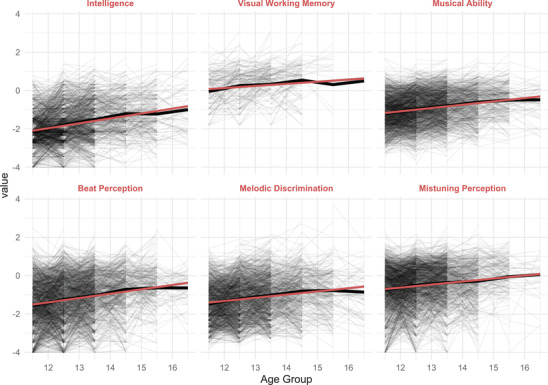
Timeline plots of cognitive and musical variables. Thick black lines represent the overall mean, and red lines represent the linear regression line. Thin gray lines represent interpolations between individual longitudinal measurements of the same individuals.

Figure 3 shows the development on the three musical and two nonmusical performance tests as well as for the aggregate variable *general musical ability* and self‐reported musical training across the year groups. The graphs show that the developmental trajectory can be reasonably well approximated by a linear regression model and deviations of the empirical means (black line) from the predicted means (red line) are generally very small and for most variables only really visible for the oldest age group.

The growth rates for all variables of interest are estimated from the longitudinal data with mixed effects models that use age as the only fixed effect. Regression coefficients for age are given in Table S2 in the supporting material. The standard deviation of the outcomes of the variables is approximately 1 due to the IRT‐scaling of the test scores, and, therefore, coefficients are approximately standardized. Results demonstrate that general intelligence, working memory, and musical abilities grow at similar rates of between 11% and 26% of a standard deviation per year. General intelligence has the largest growth rate (0.26 SD/year), and among the three musical skills, beat perception shows the largest growth (0.23 SD/year). Notably, self‐reported musical training does not show any significant development over time.

In addition to the main developmental trends, Figure 3 also shows large individual variability in developmental trajectories for each of the variables. Thin gray lines represent interpolations between individual longitudinal measurements of the same individuals. Variability is clearly visible in terms of the ability level (intercepts) as well as in terms of the change in ability across measurement points (slopes). Note that the measurements of individual trajectories reflect the true developmental change as well as noise (individual measurement error), and excessively large jumps of individual lines can be assumed to be mainly due to noise.

**Step 2. Individual differences in intraindividual change**

In this analysis step, we investigate whether some of the variability in individual developmental trajectories visible in Figure 3 can be associated with individual levels of musical training. To this end, we average the scores from the Gold‐MSI musical training subscale across all measurement points of each participant. For ease of visualization, a new categorical variable with three levels is created that characterizes participants as having a low, medium, or high musical training background. Figures S1 and S2 in the supporting material show how the average levels, as well as average developmental trajectories, differ across the three levels of musical training. For most performance scores, low musical training is associated with low levels of test scores and only moderate increases.

These associations are confirmed by the results from a series of linear mixed effects models. For each of the six outcome variables, five models with different sets of predictor variables are specified: (1) only main effect of age, (2) main effects of age and musical training, (3) main effect of age and interaction effect of age and musical training, (4) main effects of age and musical training and their interaction effect, and (5) only their interaction effect. These five model variants are compared using the BIC. The model with the lowest BIC value is selected. The model summaries in Table 1 show that for some outcome variables (i.e., visual working memory, intelligence, melodic discrimination, mistuning perception, and [general] musical ability), the interaction between age and musical training explains a significant part of the variability in addition to the main effect of age. For beat perception, the best model does not even include a main effect of age but only the interaction of age and musical training. Hence, for all outcomes, musical training is positively associated with growth in musical and cognitive skills. Note that musical training is used as a between‐participant covariate in these models.

**TABLE 1:** Regression coefficients for best linear mixed models using the variable in the first column as the dependent variable and Age Group and Mean Musical Training as independent variables

Variable	Predictor	β	95% CI	*p*
Visual Working Memory	Age Group	0.09	[0.07, 0.12]	<0.001
	Age Group x Musical Training	0.01	[0.00, 0.01]	<0.001
Intelligence	Age Group	0.21	[0.19, 0.23]	<0.001
	Age Group x Musical Training	0.01	[0.00, 0.01]	<0.001
Melodic Discrimination	Age Group	0.07	[0.05, 0.09]	<0.001
	Age Group x Musical Training	0.02	[0.02, 0.02]	<0.001
Mistuning Perception	Age Group	0.08	[0.06, 0.10]	<0.001
	Age Group x Musical Training	0.02	[0.02, 0.02]	<0.001
Beat Perception	Age Group x Musical Training	0.02	[0.02, 0.02]	<0.001
Musical Ability	Age Group	0.09	[0.07, 0.10]	<0.001
	Age Group x Musical Training	0.02	[0.02, 0.02]	<0.001

However, because musical training is used as a static and between‐participant covariate, these models only provide evidence for an association between musical training and the growth of cognitive and musical skills with no indication of whether this association is due to a directed causal effect.

**Step 3. Interrelations in behavioral change**

The graphs on intraindividual change demonstrate that cognitive and musical abilities all grow in an approximately linear fashion, and the corresponding regression models show that these abilities grow with comparable rates over time. Their regression coefficients were not identical but close enough to question whether differences might only be due to chance and thus to ask how closely change in musical and cognitive development is interrelated. Because this question concerns the broader concept of musical ability rather than individual test scores, we limit the analysis here to the aggregate variable *general musical ability* which we compare to general intelligence. Within the mixed effects framework, the question can be modeled using a multivariate mixed effects model with general musical ability and general intelligence as dependent variables and age group as an independent variable. Model comparisons allow us to test whether a model with the same slope for musical ability and general intelligence test scores is sufficient or whether different slopes are necessary to explain the change in musical ability and intelligence.

Figure S3 in the supporting material shows that the growth of general intelligence appears to be slightly stronger than the growth of general musical ability. This difference in growth rates is confirmed by a smaller BIC value (i.e., better model fit) for the mixed model with separate slopes for the growth of the two dependent variables as given in Table S4. The summary of the separate model shows that the slope for intelligence is substantially higher than the slope of general musical ability.

Hence, the development of general musical ability and intelligence is not identical and takes place at different growth rates during adolescence.

**Step 4. Causes of intraindividual change**

In this analysis step, we consider changes in concurrent musical activity (CCM) that are investigated as causes for intraindividual change. We use CCM as a dynamic predictor which means that CCM values for the same individual can change across test waves. This affords a within‐participant interpretation,^71^ which contrasts with the between‐participant interpretation presented in step 2 where we made use of the Gold‐MSI musical training subscale as a static predictor that only differs between individuals. Note that the CCM measure asks for musical activities during the 3 months prior to each testing session which makes it an antecedent to the measurements on musical and cognitive tests supporting a causal interpretation. Model selection of main effects and interaction was performed as described in step 2. Table S4 in the supporting material summarizes the selected models across all six outcome variables.

For five outcome variables, the interaction of age and concurrent musical activity is part of the best fitting model, and in all cases, this interaction has a positive coefficient. Only for beat perception, CCM does not interact with age but enters the model as the main effect. The effect sizes of the CCM interaction effect are between 0.01 and 0.07 when calculated as a difference in *R* ^2^ between models with and without the interaction effect. The largest effect of CCM is found for general musical ability, indicating that the amount of variance explained in musical ability increases by 7% if the concurrent musical activity is taken into account. Taken together, these results suggest that the age‐related increase in general musical ability is greater when adolescents have engaged in musical activities more intensely across the 3 months prior to testing, which could be cautiously interpreted as a causal effect of music activity.

**FIGURE 4:**
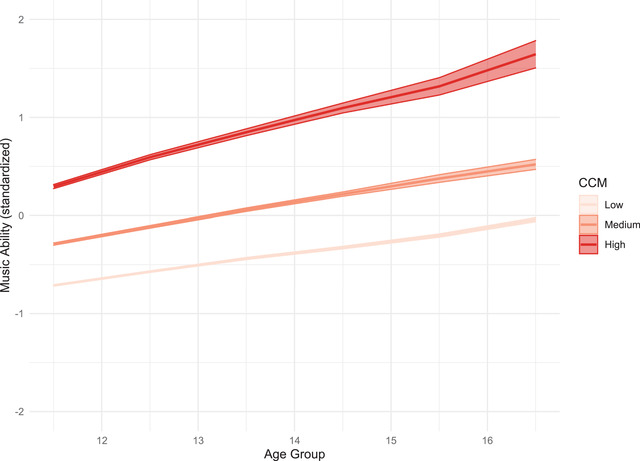
Model‐predicted growth trajectories for participants with Concurrent Musical Activities (CCM) values at the terciles of the CCM distribution.

Figure 4 gives a graphical depiction of model‐predicted growth trajectories for participants with CCM values at the terciles of the CCM distribution for which slightly different slopes are visible. The graph also shows how concurrent musical activity increases the differences in general musical ability over time.

**Step 5. Causes of interindividual differences in intraindividual change**

The causes of interindividual differences in intraindividual change can be investigated with a latent class model that aims to separate participants into different groups according to their development over time. Additional covariates can be included to predict the latent class membership. Hence, the model tries to answer the questions “Can we find distinct groups of participants who differ in their musical development over time?” and “Which variables are associated with the different growth trajectories?”

The model has a longitudinal (fixed effects) part where the dependent variable (general musical ability or intelligence) is modeled by age and the interaction of age and concurrent musical activities, following from the best model identified in step 4. In addition, the model has random effects for these same two terms as well as the general intercept which allows participants to have individual trajectories deviating from the fixed effects trends. Participants are separated into latent classes according to these same model terms (i.e., growth of musical development across age and the interaction of age and concurrent musical activity). Thus, latent classes are defined by developmental growth. Finally, class membership is explained by the level of general intelligence, working memory capacity, and musical training, which were all measured in the first year that participants entered the study. In sum, the model tries to predict musical development across adolescence based on the participants’ initial cognitive profiles and level of musical training.

Models with 1–4 latent classes are compared and the latent class model with three classes had the best fit to the data according to the BIC.

The fixed effects for the three latent classes are summarized in Table 2, which shows that class 3 has the highest intercept and largest growth across age as well as for the interaction of age and CCM. Hence, class 3 is a highly musical group that benefits strongly from musical training. In contrast, class 1 has the lowest intercept but shows relatively strong growth across age. Hence, this group seems to be falling behind with their musical abilities at the start but are able to catch up over time when compared to class 2. It is worth noting that for all three classes, the interaction of age and CCM is positive and significant.

**TABLE 2:** Model summaries for best latent class model (three classes) for Musical Ability over Age Group and CCM

Class	Term	β	Std. error	95% CI	Wald	*p*
LC1	Intercept	−2.429	0.303	[−3.023, −1.835]	−8.0	<0.001
LC2		−2.037	0.566	[−3.146, −0.929]	−3.6	<0.001
LC3		−1.473	0.247	[−1.957, −0.988]	−6.0	<0.001
LC1	Age Group	0.137	0.024	[0.091, 0.183]	5.8	<0.001
LC2		0.078	0.043	[−0.007, 0.163]	1.8	0.073
LC3		0.154	0.017	[0.120, 0.189]	8.8	<0.001
LC1	Age Group x CCM	0.011	0.003	[0.005, 0.017]	3.6	<0.001
LC2		0.012	0.005	[0.002, 0.022]	2.3	0.024
LC3		0.017	0.002	[0.013, 0.021]	9.2	<0.001

*Note*: BIC values for 1–4 number of classes are BIC(3) = 9471.5, BIC(2) = 9490.6, BIC(4) = 9499.1, and BIC(1) = 9788.5.

Abbreviations: BIC, Bayesian information criterion; CCM, concurrent musical activity.

**FIGURE 5:**
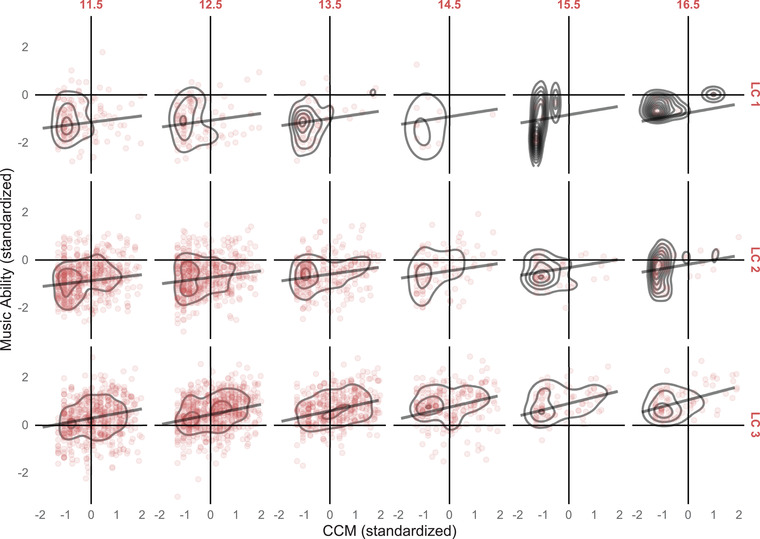
Density plot for Concurrent Musical Activities (CCM, x‐axis) versus Musical Ability (y‐axis) by Age Group and Latent Class. Black lines present model predictions. All values are standardized for better display. LC1 is the class of participants with initially the lowest levels of musical ability, and LC3 is the group with the highest initial levels of musical ability. Abbreviation: LC, latent class.

These different trajectories across age and CCM can be seen in Figure 5. The graphical display of the development of participants in class 1 (top row of Figure 5) shows an interesting pattern, where a cluster of participants with relatively high musical abilities and high concurrent musical activities separate from the rest of class 1 for the older age groups. Hence, this cluster of people seem to come from a low musical ability level but over time benefit substantially from musical activities.

**FIGURE 6:**
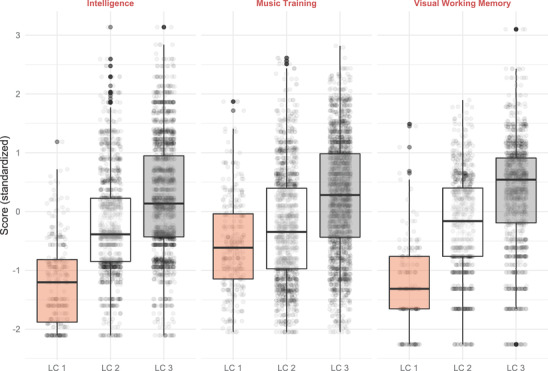
Boxplot (standardized) Intelligence, Musical Training, and Visual Working Memory scores across the three latent classes. Abbreviation: LC, latent class.

Table S5 in the supporting material shows the regression for the class membership model, demonstrating that the cognitive profile as well as the level of musical training all contribute significantly to the separation of the latent classes. The positive growth trajectory of class 1 is clearly associated with higher levels of intelligence, working memory, and musical training at study entry. The association between the membership in the three latent classes and the cognitive variables as well as musical training can also be clearly seen in the boxplot of Figure 6.

In a final step, we test whether intelligence benefits in the same way from concurrent musical activities over time by running a similar latent class model, but with general intelligence as a dependent variable and general music perception ability and musical training level at study entry as predictors for separating the two latent classes.

The model is summarized in Table S6, showing that the initial level of intelligence (intercept) and the growth of intelligence are positively related across the three groups. In other words, students with higher levels of intelligence also show the largest growth in intelligence over time. Furthermore, only the two classes with higher levels of intelligence seem to benefit significantly from the concurrent musical activity for their cognitive growth. This is in contrast to the latent class model of musical development where participants in all three classes benefitted significantly from CCM over time.

## DISCUSSION

In this study, we investigated developmental trajectories of musical skills and cognitive abilities across adolescence. The literature review in part I revealed primarily a considerable lack of quantitative longitudinal studies on musical development. In addition, the results from the few existing studies in this area seemed difficult to compare due to differences in methodology, participant samples, and age groups assessed. Part II provided insights from modeling the musical variables taken from a very large representative study. The model showed that early measures of musical ability were better predictors than measures of musical engagement. This was not only true for musical ability measured in later years but also when the later musical engagement was considered as a dependent variable. Furthermore, the model suggested that early musical encouragement by parents had a significant, but comparatively small effect, on musical abilities and engagement in later years.

Taken together, the studies reviewed in part I do not show very clear developmental trends, possibly due to small sample sizes and differences in the type of musical training intervention. Similarly, the music‐related insights gleaned from the large MCS are limited due to the sparsity and superficial nature of the musical information collected within the study. Hence, in order to fill this gap in the quantitative literature on musical development, we presented in part III results from an ongoing large‐scale study on musical and cognitive development that contains a comprehensive and dedicated battery of assessments of music perception skills and expertise, the LongGold project. The preliminary results presented here show that musical skills as well as cognitive resources grow approximately linearly with age, but individual differences in growth trajectories are substantial. This implies that any correlation between musical skills, musical training, and cognitive capacities is confounded by normal growth processes, at least during adolescence. This suggests that any far‐transfer mechanism from musical training to cognitive skills is likely to work only as a moderator, but not as a primary driver of cognitive growth. Interestingly though, musical training has a significant positive association with all musical and cognitive variables of interest. Growth rates for musical ability and intelligence are similar in our data but not identical with Intelligence growing faster at about a quarter of a standard deviation per year on average up to the age of 15 when growth rates seem to decrease.

Modeling cognitive and musical growth, we found a positive, albeit small interaction effect of concurrent musical activity and age on all musical and cognitive variables of interest. This suggests a direct causal influence, as CCM reflects musical activities which occur 3 months before taking the survey. But whether CCM is the primary cause affecting growth rates is unclear, as CCM could be just an indicator of unmeasured causes, that is, socioeconomic factors facilitating or impeding musical activity or emotionally (un)stable phases in adolescence.

In a final step, we asked whether initial levels of musical training, intelligence, and working memory can predict the future growth of musical abilities. The resulting model separated participants into distinct latent classes with different growth rates and initial levels of musical ability. The trajectories within these classes differ markedly. Participants who start with high musical ability and cognitive skills also develop their musical abilities faster. This is consistent with the findings reported by Seither‐Preissler et al. and their neurocognitive model of musical development.^18^ But the findings from the LongGold study also show a “catching up” in terms of developmental differences in the group of participants with the lowest initial levels of musical ability, though this group does not reach the (average) musical ability level of the groups with medium or high ability levels. Furthermore, density plots show that with growing age, in all three classes, a distinct cluster of active musicians develops, who also have the highest level of musical abilities within their class.

The three latent classes of musical development are very closely associated with clear profiles of both cognitive variables and musical training scores from the year that participants entered the study. Higher initial levels and faster growth of musical skills go along with higher levels of intelligence, greater working memory capacity, and stronger musical training background. This suggests that it might be possible to predict average musical development across future years from the initial profile of these three variables. Hence, these variables could be considered to be proxies for musical potential and can explain individual variability in growth trajectories. Though, it will be important to provide robust empirical confirmation in future studies using a predictive approach.

Conversely, when modeling the dependent variable intelligence with latent classes of music ability, the interaction effect of concurrent musical abilities is less strong. However, each of the latent classes had growth rates for intelligence that are proportional to their initial level of musical training. This can be indicative of a far‐transfer effect of musical training. Therefore, these results are compatible with an interpretation suggesting a stronger near‐transfer effect (from CCM to musical ability) and a weaker far‐transfer effect (from CCM to intelligence) with a size of about one‐third to one‐fourth of the stronger effect. Although it is not possible to rule out the influence of confounders, results could also reflect initial differences in the socioeconomic status where a higher status results in higher musical training levels at study entry. Future analysis of data taking into account the socioeconomic status of the participants, which is currently being collected through the ESeC inventory,^72^ could provide additional insight regarding this question.

The LongGold project is still ongoing, and the preliminary data presented here are, therefore, limited in several ways. Due to the recruitment strategy of the cohort‐sequential design, younger age groups are over‐represented in this dataset and inference is, therefore, more precise and robust for the first years of the growth trajectories. However, the confidence intervals around the upper ends of the growth curves are already reasonably narrow and the robustness and precision of the growth models in the upper teenage years will increase over the coming years as more data are collected.

The measurements on the performance tests appear to be fairly noisy as can be seen from the graphical displays of individual growth trajectories. However, the noise seen in the data can be assumed to be due to random measurement error, and noise effects seem to cancel out across individual and repeated observations which gives rise to seemingly smooth and largely linear average growth curves. Nonetheless, it is worth taking measurement error into account in future analyses and models. A principled way of incorporating measurement error can be based on the measurement error estimates generated along with the IRT ability scores of each test. While it is difficult to incorporate known measurement error at the level of the individual observation within the mixed effects model framework, future analyses making use of the structural equation model framework could take this known measurement error into account and thus make the growth models more robust.

Finally, it is necessary to acknowledge that this paper does not provide the final word on the causal relationships between musical ability and cognitive resources. Including CCM into developmental models as a dynamic within‐participant predictor allows for a causal interpretation, but only under the assumption that the causal effect indeed flows from CCM to musical ability or intelligence. This assumption is implemented in the mixed effects models but cannot be tested within these models. However, using modeling techniques from the structural equation modeling field, such as random‐intercept cross‐lagged panel models,^73^, ^74^ it may actually be possible to test whether the influence of cognitive resources on musical activities and abilities and/or the reverse relationships can be supported by longitudinal data. This approach is similar to the structural equation modeling of the MCS data presented in part II and is a primary goal for future analysis of the data collected on the LongGold project.

## AUTHOR CONTRIBUTIONS

D.M. conceived the overall manuscript. P.E. collected and analyzed the data for parts I and II and wrote the draft of these parts. D.M. conceived the analysis and wrote the draft manuscript of part III, the introduction, and the general discussion. K.F. programmed the data analysis, created tables and figures, and contributed to the writing. K.F. and D.M. edited and revised the entire manuscript.

## COMPETING INTERESTS

All three authors declare no competing interests.

### PEER REVIEW

The peer review history for this article is available at: https://publons.com/publon/10.1111/nyas.14911.

## Supporting information


**Table S1** Systematic review of longitudinal assessment of musical abilities: descriptive information on selected publications
**Table S2** Linear regression coefficients for the predictor variable Age Group for each of the seven dependent variables of interest
**Figure S1** Timeline plots for general intelligence, visual working memory, and general musical ability scores by levels of self‐reported musical training. The thick black line represents the overall mean, and thin gray lines represent individual trajectories.
**Figure S2** Timeline plots for beat perception, melodic discrimination, and mistuning perception ability scores by levels of self‐reported musical training. The thick black line represents the overall mean, and thin gray lines represent individual trajectories.
**Figure S3** Empirical and model‐based growth trajectories for intelligence and musical ability
**Table S3** Regression coefficients for standardized Intelligence and Musical Ability over age with identical or different slope coefficients
**Table S4** Mixed model summaries for all variables of interest using age and concurrent musical activity (CCM) as dynamic predictors
**Table S5** Model summary of latent class membership model for growth trajectories of Musical Ability over Age Group and CCM. Reference is Latent Class 2, the middle class
**Table S6** Model summaries for best latent class model (three classes) for Intelligence over Age Group and CCM. Note that Latent Classes are different than in the best model for Musical Ability. BIC values for models with one to four classes are BIC(3) = 9471.5, BIC(2) = 9490.6, BIC(4) = 9499.1, and BIC(1) = 9788.5

## Footnotes

a For the purpose of this paper, we generally follow the WHO's definition of adolescence as “the phase of life between childhood and adulthood, from ages 10 to 19.” See: WHO, 2022; https://www.who.int/health‐topics/adolescent‐health#tab=tab_1. However, in parts I and II, we also consider data from the childhood years as these are closely related to the adolescent data presented.

b Note that further studies on musical development^41^ that fit these search criteria but were published recently are not part of this review. Further note that some studies could not be included in the quantitative review because the means and standard deviations of the musical ability measurements for all time points were not given in the publication.^18^, ^42^

c Earlier publications based on the same longitudinal study do not offer additional information and, therefore, have not been considered further here.^45^, ^46^

d The MCS is funded by the UK's Economic and Social Research Council and a consortium of UK government departments. It is conducted by the Centre for Longitudinal Studies at University College London under the direction of Emla Fitzsimons. To our knowledge, the analysis presented here is the first analysis of the MCS data with an exclusive focus on music. Published results on many other research questions and additional information on the MCS data are available on the study's website: https://cls.ucl.ac.uk/cls‐studies/millennium‐cohort‐study/

e The LongGold Study has been funded by the Humboldt Foundation through the Anneliese‐Maier research prize awarded to Daniel Müllensiefen who is also the principal investigator of the study. The UK arm of the study is coordinated through Goldsmiths, University of London and the German branch is coordinated through the University of Music, Drama and Media, Hanover, Germany. The LongGold project is still ongoing and the current paper presents the first comprehensive and general, albeit still preliminary, longitudinal analysis of the data collected so far. However, several studies^3^, ^25^, ^57^, ^58^, ^59^ have already used data from the LongGold study to answer specific questions and more information is also available from the project's website: https://longgold.org/

f
https://longgold.org/ https://shiny.gold‐msi.org/longgold_demo/

g
https://github.com/klausfrieler/JAJ
