# Supplementary material for: Musical development during adolescence: Perceptual skills, cognitive resources, and musical training
Source: Ann N Y Acad Sci. 2022 Oct 17;1518(1):264–81. doi: 10.1111/nyas.14911 (PMC10092152; doi:10.1111/nyas.14911)
Supplement: Supplementary file 1 — Table S1 Systematic review of longitudinal assessment of musical abilities: descriptive information on selected publications Table S2 Linear regression coefficients for the predictor variable Age Group for each of the seven dependent variables of interest Figure S1 Timeline plots for general intelligence, visual working memory, and general musical ability scores by levels of self‐reported musical training. The thick black line represents the overall mean, and thin gray lines represent individual trajectories. Figure S2 Timeline plots for beat perception, melodic discrimination, and mistuning perception ability scores by levels of self‐reported musical training. The thick black line represents the overall mean, and thin gray lines represent individual trajectories. Figure S3 Empirical and model‐based growth trajectories for intelligence and musical ability Table S3 Regression coefficients for standardized Intelligence and Musical Ability over age with identical or different slope coefficients Table S4 Mixed model summaries for all variables of interest using age and concurrent musical activity (CCM) as dynamic predictors Table S5 Model summary of latent class membership model for growth trajectories of Musical Ability over Age Group and CCM. Reference is Latent Class 2, the middle class Table S6 Model summaries for best latent class model (three classes) for Intelligence over Age Group and CCM. Note that Latent Classes are different than in the best model for Musical Ability. BIC values for models with one to four classes are BIC(3) = 9471.5, BIC(2) = 9490.6, BIC(4) = 9499.1, and BIC(1) = 9788.5 [file NYAS-1518-264-s001.docx]

Supporting Material for *Musical development during adolescence: Perceptual skills, cognitive resources and musical training*

Daniel Müllensiefen, Paul Elvers, Klaus Frieler

# Part I: Systematic review of longitudinal studies on musical development

| **Table S1** Systematic Review of Longitudinal Assessment of Musical Abilities: Descriptive Information on Selected Publications | | | | | | | | | | |
| --- | --- | --- | --- | --- | --- | --- | --- | --- | --- | --- |
| ID | Citation | Intervention (Y/N) | Intervention Type | Control Group | Participant Age Start | Study Duration | Measure | Interval | N | Group Factor |
| 1 | Hassler (1992) | No | NA | NA | 11 | 8 years | Wing Musical Inteligence | yearly | 120 | Gender |
| 2 | Hassler and Birbaumer (1988) | No | NA | NA | 11 | 5 years | Wing Musical Inteligence | yearly | 120 | Gender & Musicality |
| 3 | Ilari et al. (2016) | Yes | El Sistema-Inspired Program | Yes | 6, 5 years | 1 year | PMMA | 1 year | 50 | Gender |
| 4 | Yang et al. (2014) | Yes | Music training | Yes | 7 years | 5 years | Music Achievement Test | six months | 250 | Musicality |
| 5. | Cohrdes et al. (2018) | Yes | Music Training | Yes | 5,5 years | 6 months | PMMA | six months | 202 | Musicality, Active Control |

# Part III: The development of musical skills, working memory, and general intelligence during adolescence

## Intraindividual change

**Table S2** Linear regression coefficients for the predictor variable Age Group for each of the seven dependent variables of interest

| Variable | beta | 95% CI | p |
| --- | --- | --- | --- |
| Intelligence | 0.26 | [0.24, 0.27] | < .001 |
| Visual Working Memory | 0.11 | [0.09, 0.14] | < .001 |
| Musical Ability | 0.17 | [0.16, 0.19] | < .001 |
| Beat Perception | 0.23 | [0.21, 0.25] | < .001 |
| Melodic Discrimination | 0.17 | [0.15, 0.19] | < .001 |
| Mistuning Perception | 0.16 | [0.14, 0.18] | < .001 |
| Musical Training | 0.02 | [-0.00, 0.04] | .118 |

## 2. Individual differences in intraindividual change

**Figure S1** Timeline plots for general intelligence, visual working memory and general musical ability scores by levels of self-reported musical training. Thick black line represents overall mean, thin grey lines represent individual trajectories.


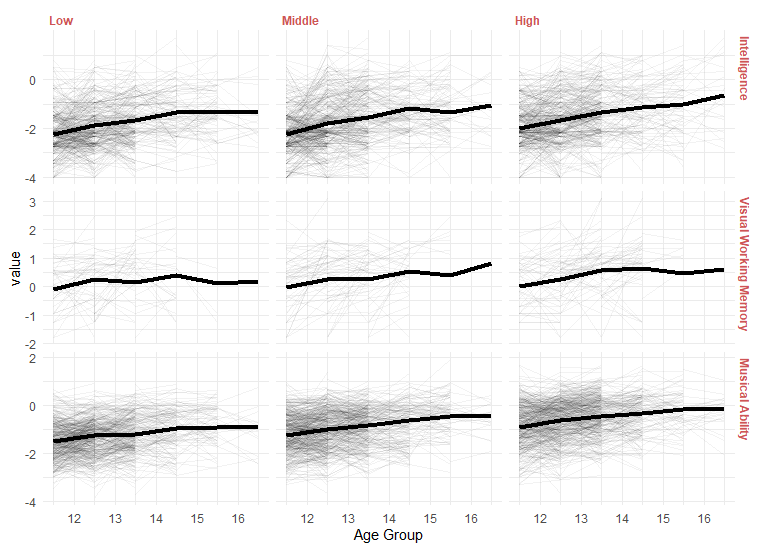


**Figure S2** Time-line plots for beat perception, melodic discrimination and mistuning perception ability scores by levels of self-reported musical training. Thick black line represents overall mean, thin grey lines represent individual trajectories.


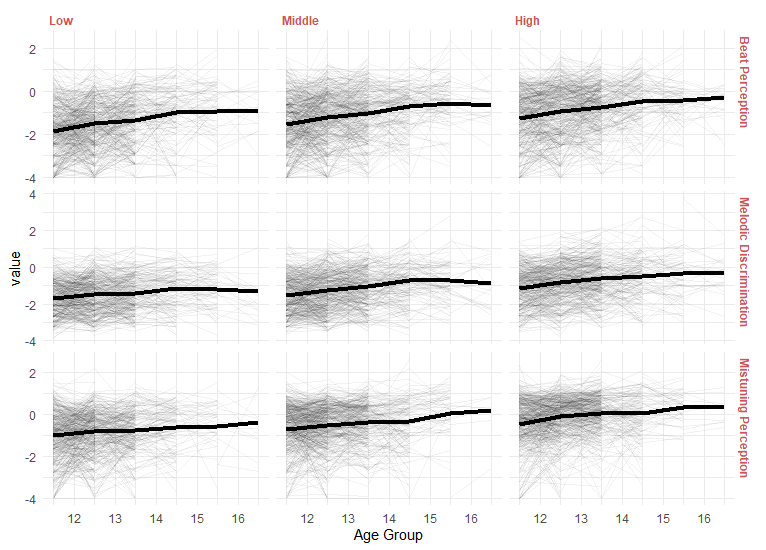


## 3. Interrelations in behaviorial change.

**Figure S3** Empirical and model-based growth trajectories for intelligence and musical ability


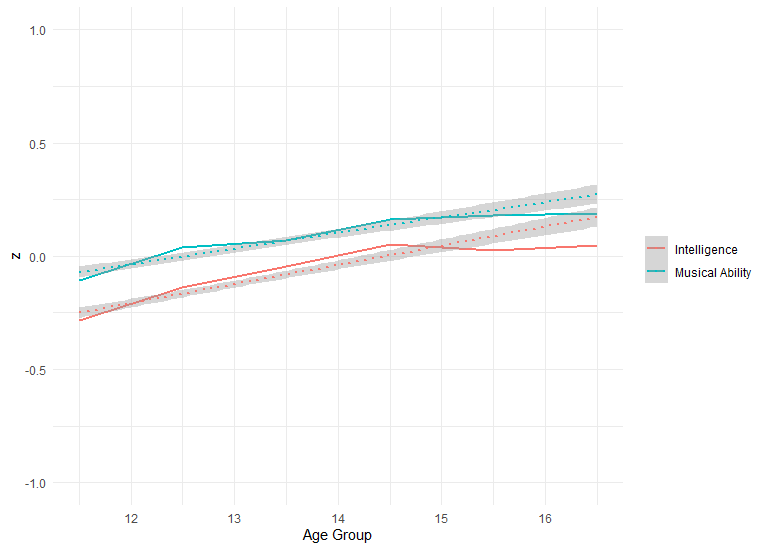


**Table S3** Regression coefficients for standardized Intelligence and Musical Ability over age with identical or different slope coefficients

| Model | BIC | term | beta | 95% CI |
| --- | --- | --- | --- | --- |
| Single slope | 33051 | Intelligence + Music Ability | 0.18 | [0.17, 0.19] |
| Different slopes | 33037 | Intelligence | 0.21 | [0.19, 0.23] |
|  |  | Music Ability | 0.15 | [0.14, 0.17] |

## 4. Causes of intra-individual change

**Table S4** Mixed model summaries for all variables of interest using age and concurrent musical activity (CCM) as dynamic predictors

| Variable | term | beta | 95% CI | p | ΔR^2^ marg. |
| --- | --- | --- | --- | --- | --- |
| Visual Working Memory | Age Group | 0.120 | [0.095, 0.145] | < .001 | .0127 |
|  | Age Group x CCM | 0.004 | [0.002, 0.005] | < .001 |  |
| Intelligence | Age Group | 0.249 | [0.232, 0.267] | < .001 | .0132 |
|  | Age Group x CCM | 0.005 | [0.004, 0.006] | < .001 |  |
| Melodic Discrimination | Age Group | 0.153 | [0.134, 0.173] | < .001 | .0562 |
|  | Age Group x CCM | 0.010 | [0.009, 0.011] | < .001 |  |
| Mistuning Perception | Age Group | 0.163 | [0.142, 0.183] | < .001 | .0451 |
|  | Age Group x CCM | 0.008 | [0.007, 0.009] | < .001 |  |
| Beat Perception | Age Group | 0.225 | [0.204, 0.247] | < .001 | .0293 |
|  | CCM | 0.097 | [0.081, 0.112] | < .001 |  |
| Musical Ability | Age Group | 0.163 | [0.149, 0.177] | < .001 | .0669 |
|  | Age Group x CCM | 0.008 | [0.007, 0.009] | < .001 |  |

## 5. Causes of interindividual differences in intraindividual change

**Table S5** Model summary of latent class membership model for growth trajectories of Musical Ability over Age Group and CCM. Reference is Latent Class 2, the middle class

| Class | Term | Beta | Std. Error | 95% CI | Wald | p |
| --- | --- | --- | --- | --- | --- | --- |
| LC3 | Intercept | -1.619 | 0.607 | [-2.809, -0.430] | -2.7 | .008 |
| LC1 |  | -0.213 | 0.373 | [-0.943, 0.518] | -0.6 | .568 |
| LC3 | Intelligence | 0.583 | 0.123 | [0.342, 0.823] | 4.7 | < .001 |
| LC1 |  | -0.343 | 0.096 | [-0.530, -0.155] | -3.6 | < .001 |
| LC3 | Mean Musical Training | 0.471 | 0.122 | [0.232, 0.710] | 3.9 | < .001 |
| LC1 |  | -0.316 | 0.097 | [-0.505, -0.127] | -3.3 | .001 |
| LC3 | Visual Working Memory | 0.287 | 0.170 | [-0.046, 0.621] | 1.7 | .091 |
| LC1 |  | -0.654 | 0.106 | [-0.861, -0.446] | -6.2 | < .001 |

**Table S6** Model summaries for best latent class model (three classes) for Intelligence over Age Group and CCM. Note that Latent Classes are different than in the best model for Musical Ability. BIC values for models with one to four classes are BIC(3) = 9471.5, BIC(2) = 9490.6, BIC(4) = 9499.1, BIC(1) = 9788.5

| Class | Term | Beta | Std. Error | 95% CI | Wald | p |
| --- | --- | --- | --- | --- | --- | --- |
| 1 | Intercept | -4.358 | 0.169 | [-4.690, -4.027] | -25.8 | < .001 |
| 2 |  | -4.417 | 0.531 | [-5.457, -3.377] | -8.3 | < .001 |
| 3 |  | -4.332 | 0.305 | [-4.929, -3.735] | -14.2 | < .001 |
| 1 | Age Group | 0.186 | 0.013 | [0.160, 0.212] | 13.9 | < .001 |
| 2 |  | 0.132 | 0.044 | [0.046, 0.218] | 3.0 | .003 |
| 3 |  | 0.278 | 0.023 | [0.233, 0.322] | 12.2 | < .001 |
| 1 | Age Group x CCM | 0.004 | 0.001 | [0.002, 0.005] | 4.9 | < .001 |
| 2 |  | -0.002 | 0.002 | [-0.006, 0.002] | -1.1 | .286 |
| 3 |  | 0.004 | 0.001 | [0.001, 0.006] | 2.6 | .008 |
